# The diagnostic performance of the one-step nucleic acid amplification assay for the detection of sentinel lymph node metastases in cytokeratin 19-positive breast cancer: a PRISMA-compliant meta-analysis

**DOI:** 10.3389/fmed.2024.1391621

**Published:** 2024-09-09

**Authors:** Meirong Liu, Weihua Wang, Yufang Wang

**Affiliations:** ^1^Department of Oncology, Liaocheng People's Hospital, Liaocheng, Shandong, China; ^2^Department of Central Laboratory, Liaocheng People's Hospital, Liaocheng, Shandong, China; ^3^Department of Radiotherapy, Liaocheng People's Hospital, Liaocheng, Shandong, China

**Keywords:** OSNA, CK19, breast cancer, sentinel lymph nodes, metastasis, meta-analysis

## Abstract

**Background:**

The status of the sentinel lymph nodes (SLNs) is an important prognostic factor for many different types of cancer. The one-step nucleic acid amplification (OSNA) assay has emerged as a rapid intraoperative molecular diagnostic tool for LN metastasis detection. We aimed to evaluate and summarize the value of the OSNA assay for the diagnosis of SLN metastasis in cytokeratin 19 (CK19)-positive breast cancer.

**Methods:**

To evaluate the diagnostic value, the sensitivity, specificity, positive likelihood ratio (PLR), negative likelihood ratio (NLR), diagnostic odds ratio (DOR), and area under the curve (AUC) were pooled. The threshold effect, followed by subgroup analysis, was performed to explore the source of heterogeneity. A sensitivity analysis was performed to assess the stability of this meta-analysis model. Fagan plots and likelihood ratio scattergrams were used to explore the potential clinical significance.

**Results:**

A total of 29 eligible studies, which consisted of 5,331 patients with 10,343 SLNs, were included in this meta-analysis. The pooled sensitivity, specificity, PLR, NLR, and DOR were 0.86 (95% CI: 0.85–0.88), 0.94 (95% CI, 0.94–0.95), 18.00 (95% CI, 13.54–23.92), 0.13 (95% CI, 0.10–0.17), and 138.99 (95% CI, 86.66–222.92), respectively. The AUC was 0.97 (95% CI, 0.95–0.98). Sensitivity analysis showed that four studies had an impact on the pooled results and mainly contributed to the heterogeneity. Fagan's nomogram revealed that the prior probability was 50%, the post-probability positive was 95%, and the post-probability negative was 11%.

**Discussion:**

Our results suggested that OSNA can predict the occurrence of SLN metastasis in CK19-positive breast cancer. However, more well-designed and multicenter diagnostic tests are needed to validate our results.

## 1 Introduction

Breast cancer is the most common malignancy in women worldwide and is a leading cause of death for women in their 40's (1). Approximately one-third of patients with primary breast cancer eventually develop distant metastases and succumb to the disease (2). The spread of tumor cells to SLNs is still an important prognostic factor for patients with breast cancer and a key criterion for determining individual treatment plans. The nodal status quantifies the number, locations, or size of involved LNs with metastases in clinical tumor node metastasis (TNM) staging of breast cancer patients (2, 3). A SLN biopsy is the standard procedure used to accurately stage axillary nodal involvement in early-stage breast cancer patients without clinical evidence of node metastasis (4). During SLN biopsy, a reliable intraoperative examination of SLN plays an important role in the decision-making process, which may help in selecting a surgical procedure or conservative treatment of the axilla in a single procedure (5). Intraoperative hematoxylin and eosin (H&E) pathological examinations of the frozen section (FS) or imprint cytology are recommended (6). However, all these methods have some drawbacks, such as inaccuracy and long required time, and do not offer timely intraoperative SLN evaluation. Therefore, there is an urgent need for a more efficient method for intraoperative detection of SLN metastasis in CK19-positive breast cancer.

The OSNA assay has emerged as a rapid intraoperative molecular diagnostic tool for lymph node metastasis (LNM) detection (7). It is a standardized and observer-independent molecular technique that can detect tumor-specific CK19 mRNA and is widely used in hospitals (8). CK19, a member of the keratin family, is widely used as an epithelial marker in clinical applications and has been used as a useful tool in the diagnosis, treatment, and prognosis of tumors (9). CK19 is one of the main cytoskeleton proteins in epithelial cells, which is released as a full-length protein by viable epithelial tumor cells and is associated with metastatic progression in cancer patients (10). To date, numerous preliminary and multicenter clinical studies have confirmed that the OSNA assay can be applied to evaluate LNM in various cancers expressing CK19, such as breast cancer, cervical and endometrial cancer, lung cancer, gastric cancer, and colorectal cancer (8, 11–13). The comparative studies between OSNA and pathological evaluation for detecting LNM in breast cancer showed that OSNA has high specificity (94.8%), a high concordant rate (93.8%), and a negative predictive value (97.6%) in a pooled assessment (13). Similar results have been found in multicenter studies for gastric cancer, colorectal cancer, and lung cancer (13). The OSNA assay is a quick and semiautomatic procedure and can be completed in ~40 min, making it suitable as an intraoperative procedure for the detection of LNM (14). Importantly, the OSNA assay is more objective, sensitive, and accurate compared with routine histopathological examination (15, 16). To date, many studies have focused on the diagnostic value of the OSNA assay for SLN detection in breast cancer, but the results are still uncertain. The expected drawbacks of the OSNA method are the false-negative results caused by unstable CK19 expression.

To the best of our knowledge, although some studies have performed intraoperative evaluation for SLN metastases using the OSNA assay in invasive breast cancer patients, there is no systematic review or meta-analysis about the value of the OSNA assay in breast cancer diagnosis. Therefore, we aimed to conduct a meta-analysis to evaluate and summarize the value of the OSNA assay for the diagnosis of SLN metastasis in CK19-positive breast cancer patients.

## 2 Materials and methods

### 2.1 Literature search strategy

We conducted this meta-analysis according to the guidelines of the Preferred Reporting Items for Systematic Reviews and Meta-Analyses (PRISMA). A systematic search was conducted in PubMed, Cochrane Library, and Web of Science to identify all potential literature, with a search period up to 1 September 2023. In addition, we supplemented the manual search to find relevant literature. The search strategy was performed by two investigators independently. Quality studies were needed to provide information on the diagnostic accuracy of OSNA for SLN metastasis in patients with breast cancer. Subject terms used for the literature search included “molecular intraoperative” or “intraoperative molecular analysis” or “intraoperative nucleic acid amplification” or “one step nucleic acid amplification” or “one-step nucleic acid amplification” or “OSNA” combined with “breast cancer” or “breast neoplasm” or “breast carcinoma” or “breast tumor.” No further ethical approval is required since the program does not require the recruitment of patients or the collection of personal information. The review of this meta-analysis has not been registered with PROSPERO or INPLASY.

### 2.2 Literature selection criteria

All articles were screened according to the inclusion and exclusion criteria by two independent reviewers (Meirong Liu and Weihua Wang). Disagreements were adjudicated by the third reviewer (Yufang Wang). The inclusion criteria were as follows: (1) patients who were diagnosed with breast cancer; (2) the specimens collected were fresh SLNs; (3) the study's purpose was to investigate the performance of the OSNA assay for detecting SLN metastasis in breast cancer patients; (4) the reference method for detecting SLN metastasis was postoperative pathology; (5) the study adopted identical machines and thresholds recommended by the OSNA manufacturer, Sysmex company; (6) the method of pathological examination was described in detail; (7) the study analysis was based on per node; and (8) extracted data were available for obtaining true-positive (TP), false-positive (FP), false-negative (FN), and true-negative (TN) values. The exclusion criteria were as follows: (1) non-English articles; (2) non-clinical research literature, including basic experiments, reviews, conference abstracts, and letters to journal editors; and (3) intraoperative pathologies, such as FS or touch imprint cytology (TIC) (17).

### 2.3 Data extraction and quality assessment

Two researchers extracted the information and assessed the quality of the included studies. The data extracted included the details on the first author, year of publication, country, type of study design, number of patients, number of LNs, number of study centers, section interval, reference standard method, and type of samples. Diagnostic accuracy estimates included TP, TN, FPs, and FN. The quality of the diagnostic studies was assessed using the Quality Assessment of Diagnostic Accuracy Studies 2 (QUADAS-2) (18). Different opinions will be settled through group consultation.

### 2.4 Statistical analyses

All analyses were performed with STATA 16.0, RevMan 5.2, and Meta-Disc 1.4 software (19). Heterogeneity was assessed using Higgin's *I*^2^ and Cochran's Q tests. *I*^2^ > 50% was considered a significant heterogeneity (20). Subgroup analyses were performed to uncover the source of heterogeneity. Sensitivity analysis was used to assess the stability of the model. The diagnostic accuracy of OSNA for SLN metastasis was quantified by the area under the summary receiver operating characteristic curve (AUC), summary DOR, summary sensitivity, specificity, PLR, NLR, and their 95% confidence interval (CI). Deeks' funnel plot was conducted to assess publication bias (21, 22). To explore the potential clinical significance, the Fagan plot was drawn to reveal the relevance between pre-test probability, post-test probability, and likelihood ratio. Moreover, we generated a likelihood ratio scattergram, which showed the different diagnostic values of OSNA for SLN metastasis in CK19-positive breast cancer patients. All statistical tests were two-sided, and a *P*-value of < 0.05 was considered significant unless otherwise indicated.

## 3 Results

### 3.1 Literature search

**Figure 1:**
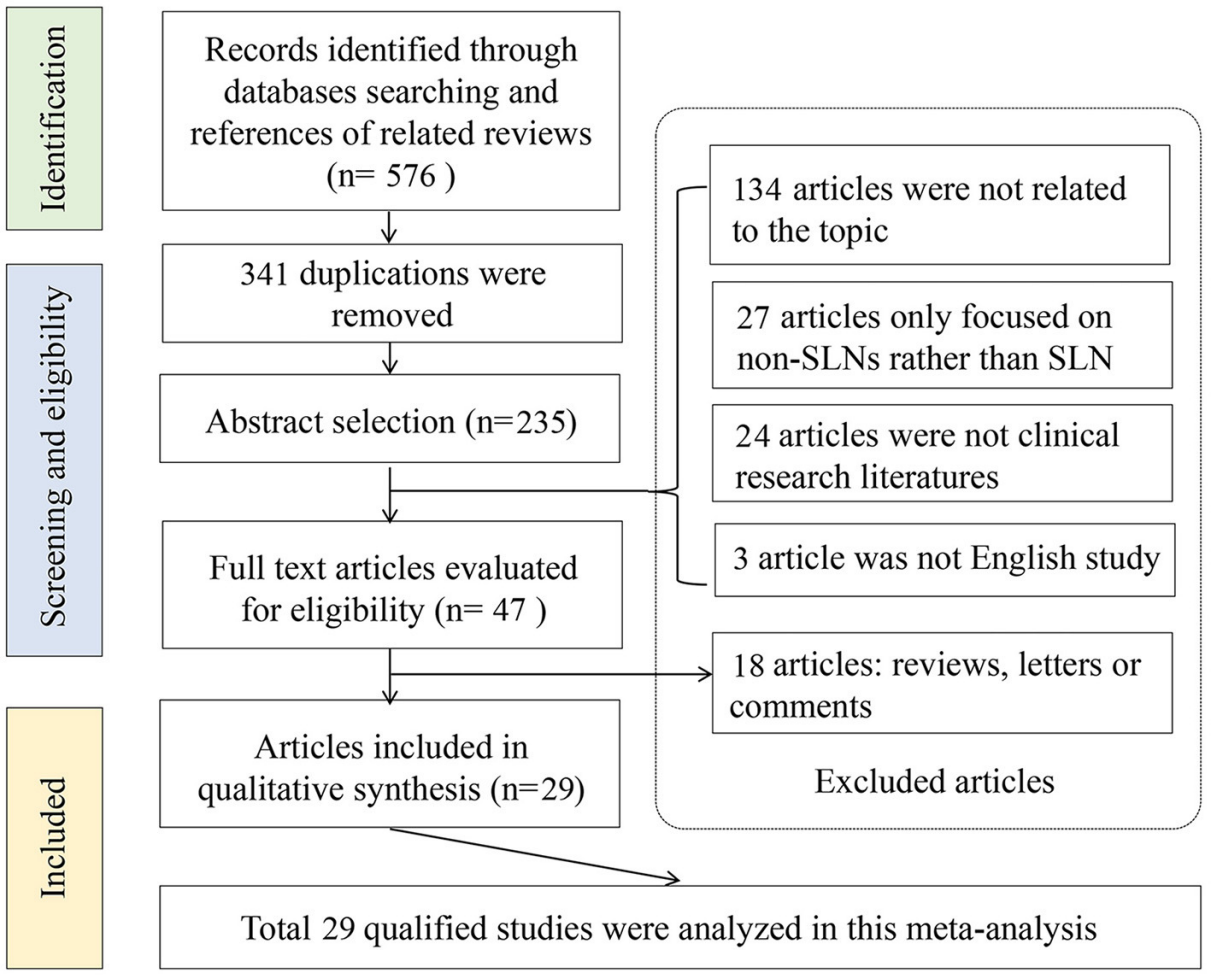
Flow chart of literature screening.

An initial literature search yielded 576 potential articles from three databases. After excluding duplicate publications, 235 studies remained. After browsing the titles and abstracts, studies were also excluded because 134 articles were not related to the topic, 27 articles only focused on the non-SLNs, and 24 articles were not clinical studies. Then, the 47 remaining studies were further evaluated through full-text reading. A total of 18 articles were further excluded due to insufficient data for diagnostic testing. Finally, 29 articles involving 5,331 patients were included in the meta-analysis (5, 14, 15, 23–48). The flow chart of the literature screening process is shown in Figure 1.

### 3.2 Characteristics of the included studies

The basic characteristics of the 29 included studies (5, 14, 15, 23–47) are shown in Table 1. Our study consisted of 5,331 patients with 10,343 SLNs. The literature was published from 2008 to 2023; 15 literature articles (7, 11, 21, 26, 28, 30, 31, 35, 37–42, 44) were conducted in Asian countries, 11 trials (5, 19, 20, 22, 23, 25, 27, 29, 32, 34, 43) were conducted in Europe, two trials (33, 36) were conducted in Oceania, and one study (24) was conducted in America. The reference standards of all studies were assessed by postoperative pathology, but the detailed approaches were different because 21 studies (7, 11, 19–28, 31–36, 38, 40, 41, 43) were taken with serial sections with HE staining and IHC and seven studies (5, 29, 30, 37, 39, 42, 44) were taken with serial sections with HE staining only.

**Table 1:** Basic characteristics of all eligible studies in this meta-analysis.

**References**	**Country**	**Reference method**	**No. patients**	**No. SLNs**	**TP**	**FP**	**FN**	**TN**
Banerjee et al. (36)	UK	HE and IHC	170	268	39	10	2	217
Bernet et al. (26)	Spain	HE and IHC	185	181	42	1	0	138
Bettington et al. (37)	Australia	HE and IHC	35	63	9	3	1	52
Buglioni et al. (33)	Italy	HE	709	903	174	28	14	687
Chaudhry et al. (38)	UK	HE and IHC	54	166	13	17	1	135
Feldman et al. (28)	America	HE and IHC	496	1,044	107	38	31	868
Goda et al. (48)	Japan	HE	65	312	53	10	8	241
Hao et al. (43)	China	HE	102	175	39	13	9	113
Inua et al. (5)	UK	HE	691	684	44	58	10	572
Jara-Lazaro et al. (39)	Singapore	HE and IHC	54	98	15	5	3	75
Khaddage et al. (27)	France	HE and IHC	46	80	15	1	2	62
Le Frère-Belda et al. (31)	France	HE and IHC	234	503	51	27	12	413
Li et al. (15)	China	HE and IHC	115	311	30	9	6	266
Osako et al. (34)	Japan	HE	80	307	53	20	7	222
Pathmanathan et al. (40)	Australia	NA	98	170	25	5	3	137
Pina et al. (47)	France	HE and IHC	197	197	30	44	10	113
Sagara et al. (35)	Japan	HE and IHC	53	61	9	1	3	48
Schem et al. (24)	Germany	HE and IHC	93	343	105	25	4	209
Shigematsu et al. (45)	Japan	HE and IHC	499	1,103	104	26	30	943
Shimazu et al. (46)	Japan	HE	63	150	63	1	3	83
Snook et al. (29)	UK	HE and IHC	194	395	66	10	6	313
Sun et al. (30)	China	HE and IHC	90	189	32	4	4	149
Takamoto et al. (44)	Japan	HE and IHC	88	300	18	8	6	83
Tamaki et al. (25)	Japan	HE and IHC	198	574	89	25	11	449
Terada et al. (41)	Japan	HE	89	111	10	3	14	94
Tsujimoto et al. (14)	Japan	HE and IHC	49	81	14	1	2	64
Visser et al. (23)	Netherlands	HE and IHC	32	346	61	15	3	267
Wang et al. (32)	China	HE and IHC	552	1,188	159	71	31	927
Wang et al. (42)	Singapore	HE and IHC	NA	40	19	0	1	20

No., Number of; SLN, sentinel lymph nodes; HE, hematoxylin and eosin; IHC, immunohistochemistry; TP, true positive; TN, true negative; FP, false positive; FN, false negative; NA, not available.

### 3.3 Quality assessment

**Figure 2:**
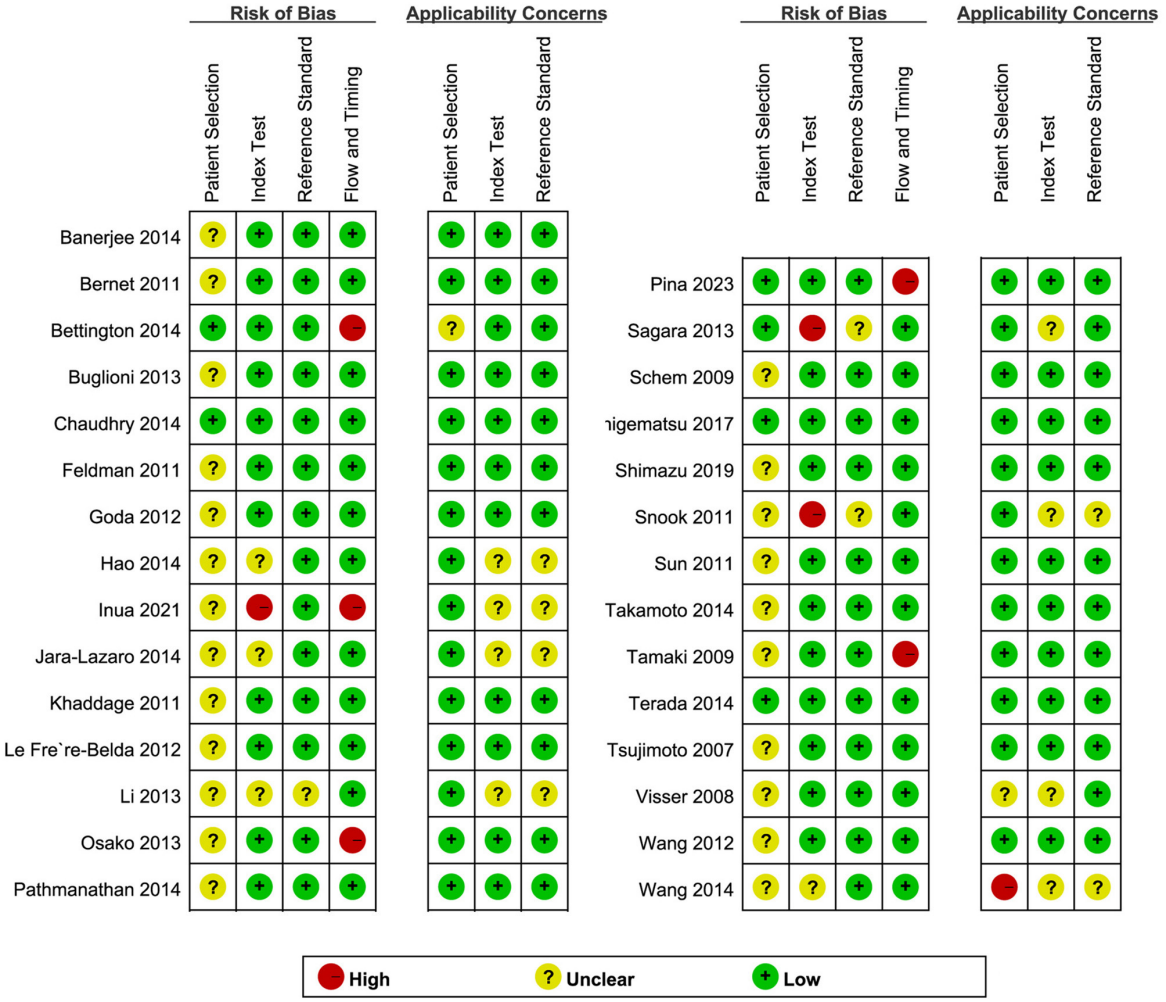
The methodological quality of individual studies.

QUADAS-2 was used to assess the quality of the included literature (Figure 2). In the assessment of the index test, three studies (5, 25, 31) were found to have a high risk of bias, and the remainder had an unclear or low risk of bias. With regard to the flow and timing domain, five studies (5, 21, 30, 33, 43) were high risk and the rest were unclear or low risk. This is mainly related to the unclear implementation of literature blinding and reporting of thresholds and loss to follow-up. In the remaining two QUADAS-2 domains, namely, patient selection and reference standard, most studies were found to be of unclear or low risk. Regarding applicability concerns, only one study (38) showed high risk in “patient selection” and no high risk in the “index test” or the “reference standard.” Overall, the quality of the studies included in this review was indicated to be acceptable.

### 3.4 Diagnostic accuracy of OSNA for SLNs in breast cancer

#### 3.4.1 Sensitivity and specificity

**Figure 3:**
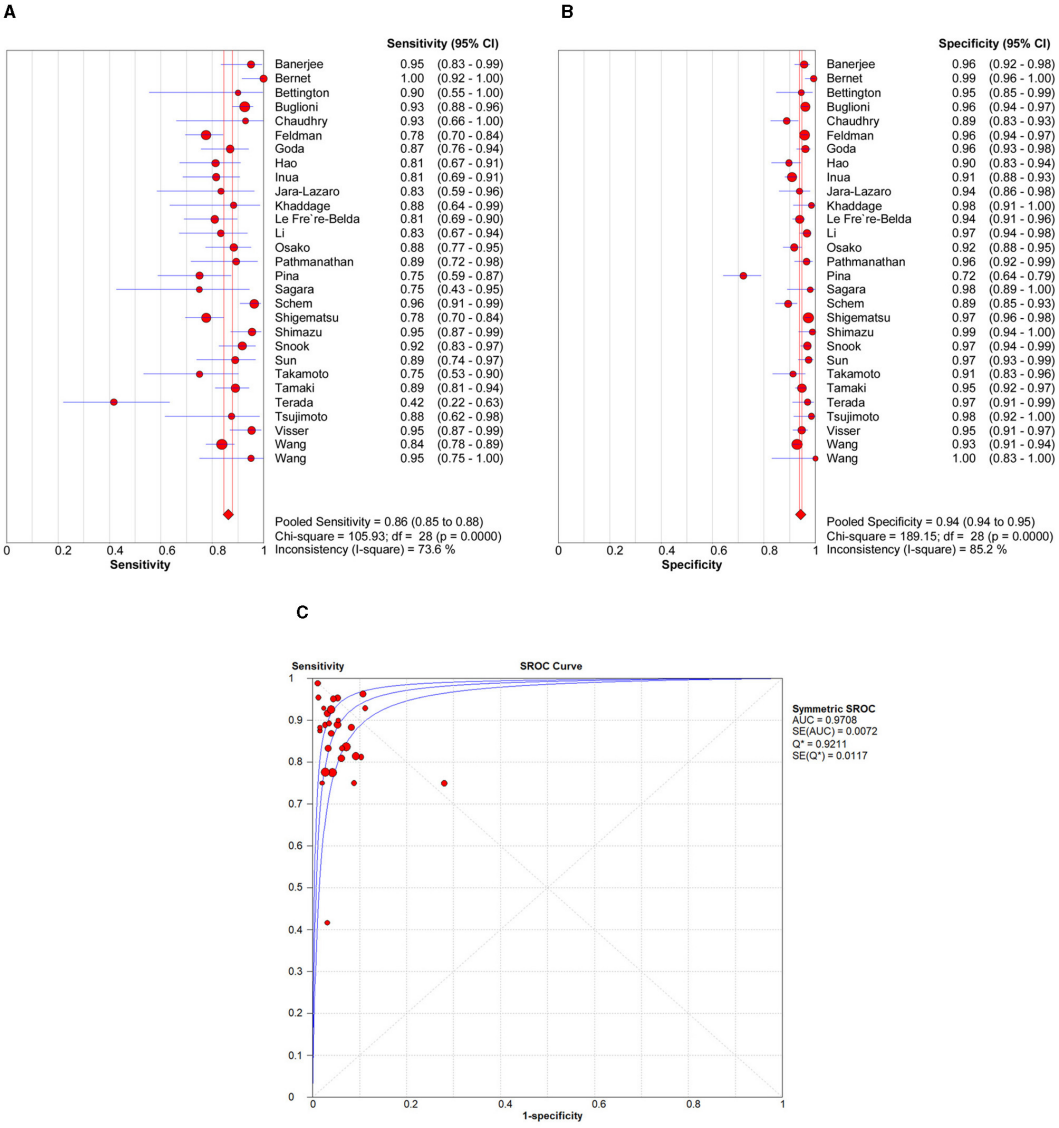
Forest plot of the sensitivity **(A)**, specificity **(B)**, and SROC curve **(C)** of OSNA for SLN metastasis in breast cancer. SROC, summary receiver operating characteristic; AUC, area under the curve; OSNA, one-step nucleic acid amplification; SLNs, sentinel lymph nodes.

The pooled sensitivity and specificity of OSNA to diagnose SLN metastasis in CK19-positive breast cancer were 0.86 (95% CI from 0.85 to 0.88) and 0.94 (95% CI from 0.94 to 0.95), respectively (Figures 3A, B). Cochran *Q* and *I*^2^-tests were conducted to assess heterogeneity, which indicated significant heterogeneity (*I*^2^ = 73.6%, *P* < 0.001) and specificity (*I*^2^ = 85.2%, *P* < 0.001). Thus, a random effect model was used.

#### 3.4.2 Summary receiver operating characteristic (SROC) curve analysis

An SROC curve analysis was performed, and the AUC of the SROC curve was calculated to be 0.9708 (95% CI from 0.95 to 0.98; Figure 3C). This indicates the high diagnostic value of SLN metastasis for CK19-positive breast cancer.

#### 3.4.3 Positive and negative likelihood ratios (PLR and NLR)

**Figure 4:**
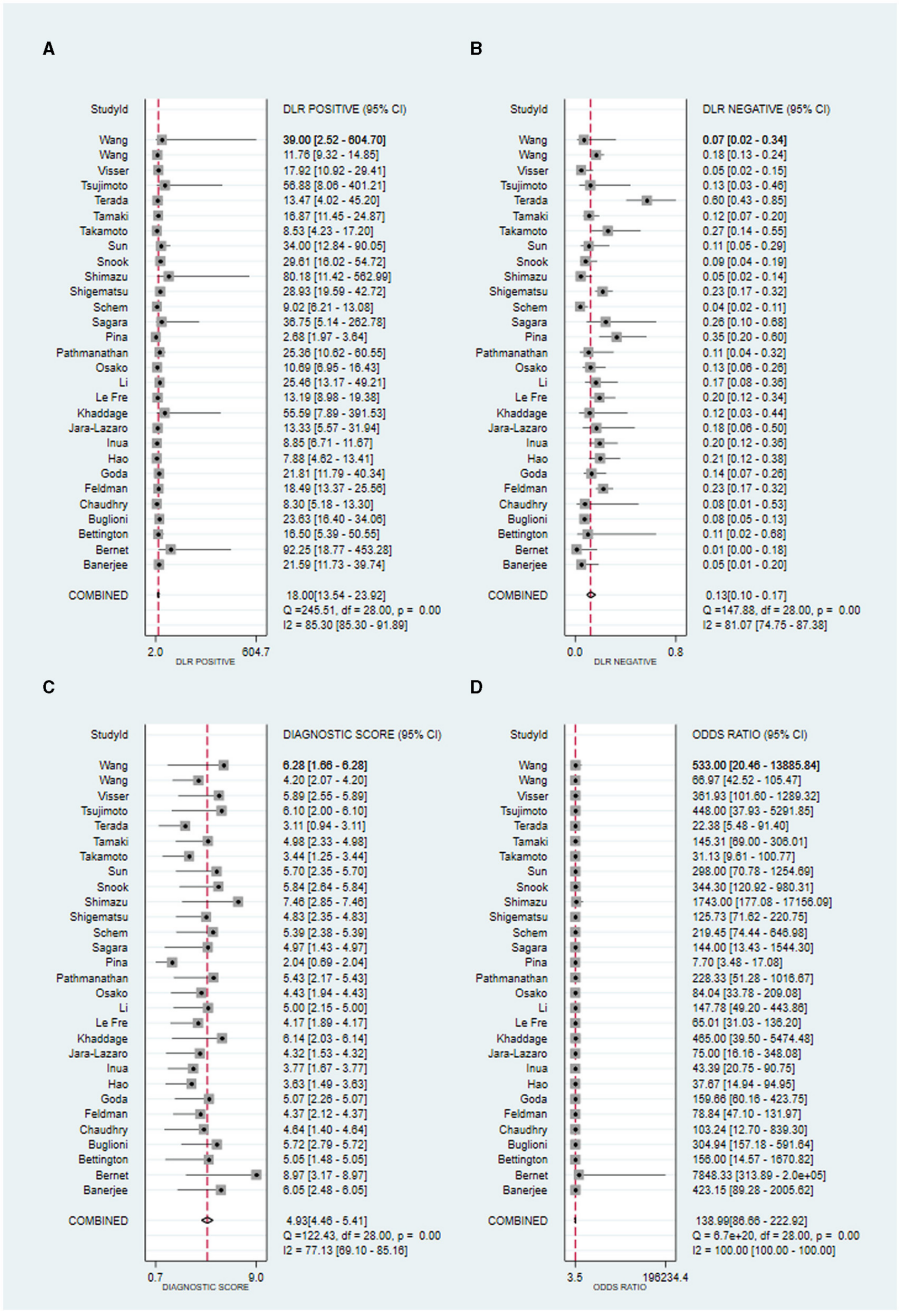
Forest plot of the PLR **(A)**, NLR **(B)**, DS **(C)**, and DOR **(D)** of OSNA for SLN metastasis in breast cancer. PLR, positive likelihood ratio; NLR, negative likelihood ratio; DS, diagnostic score; DOR, summary diagnostic odds ratio; OSNA, one-step nucleic acid amplification; SLNs, sentinel lymph nodes.

Owing to significant heterogeneity in PLR (*I*^2^ = 85.30%, 95% CI from 85.30 to 91.89, *P* < 0.001) and NLR (*I*^2^ = 81.07%, 95% CI from 74.75 to 87.38, *P* < 0.001), meta-analyses were performed using a random-effects model. The pooled PLR and NLR of the studies were 18.00 (95% CI from 13.54 to 23.92) and 0.13 (95% CI from 0.10 to 0.17), respectively (Figures 4A, B).

#### 3.4.4 Diagnostic score (DS) and odds ratio (DOR)

As there was significant heterogeneity in DS (*I*^2^ = 77.13%, *P* < 0.001) and DOR (*I*^2^ = 100.00%, *P* < 0.001), a meta-analysis of DS and DOR was conducted using a random-effects model. The overall pooled DS and DOR of the studies were 4.93 (95% CI: 4.46–5.41) and 138.99 (95% CI: 86.66–222.92), respectively (Figures 4C, D).

#### 3.4.5 Sensitivity analysis

**Figure 5:**
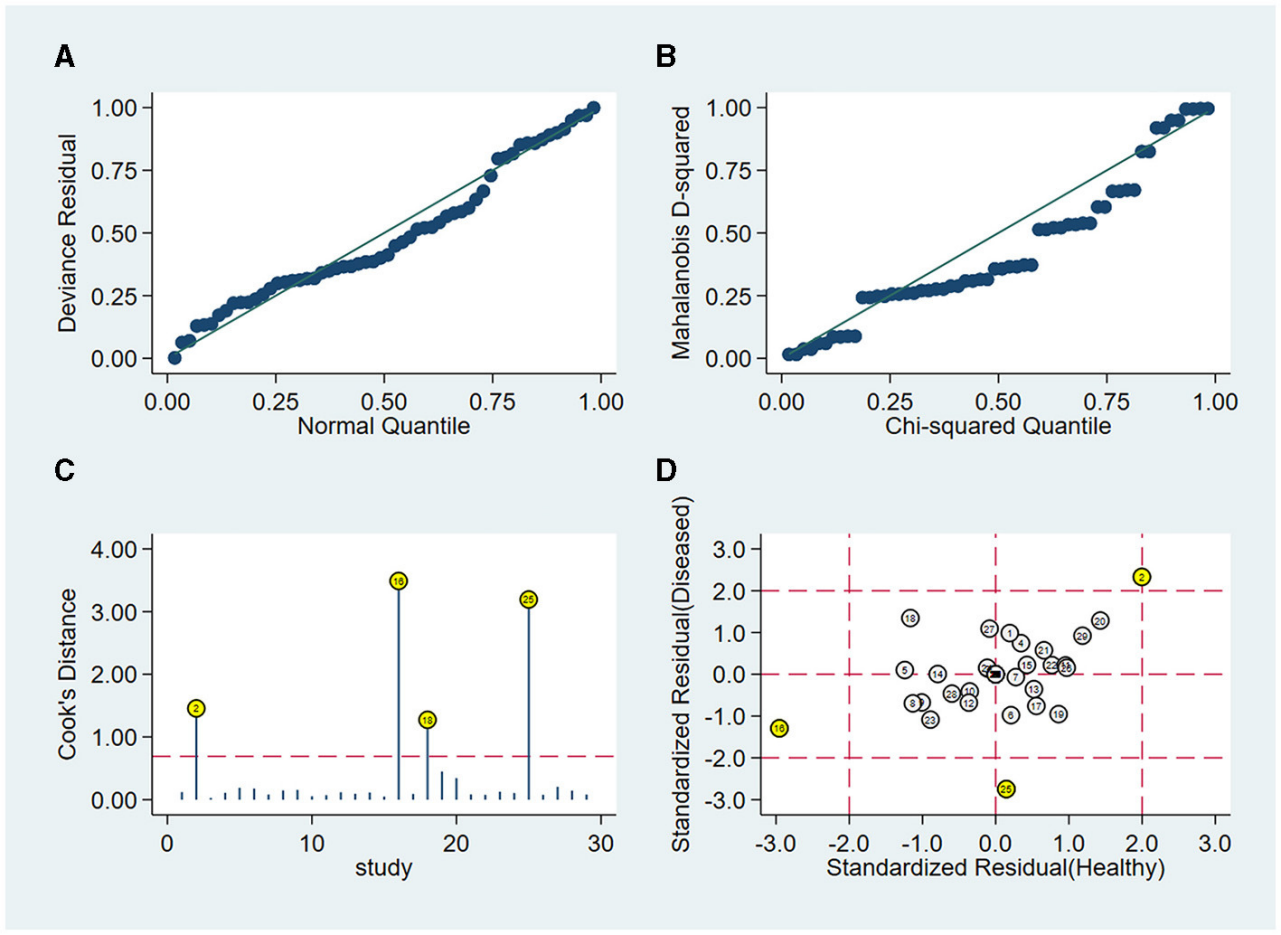
Sensitivity analysis results of the included studies. **(A)** Goodness of fit, **(B)** bivariate normality, **(C)** influence analysis, and **(D)** outlier detection.

Sensitivity analysis showed a good fit for goodness of fit and binary normality (Figures 5A, B). There were four articles weighted (Figure 5C), which may be a source of heterogeneity shown by outlier detection (Figure 5D). After the exclusion of abnormal studies, the pooled specificity varied from 0.94 to 0.95; the sensitivity, AUC value, and NLR remained unchanged; the DLR decreased slightly from 18.26 to 18.00, and the DS and DOR decreased from 4.93 to 4.91 and 138.99 to 135.57, respectively. These data suggested that the re-analysis was slightly different compared with the combined results before exclusion which indicates that the conclusions of this study are robust.

#### 3.4.6 Risk of publication bias

**Figure 6:**
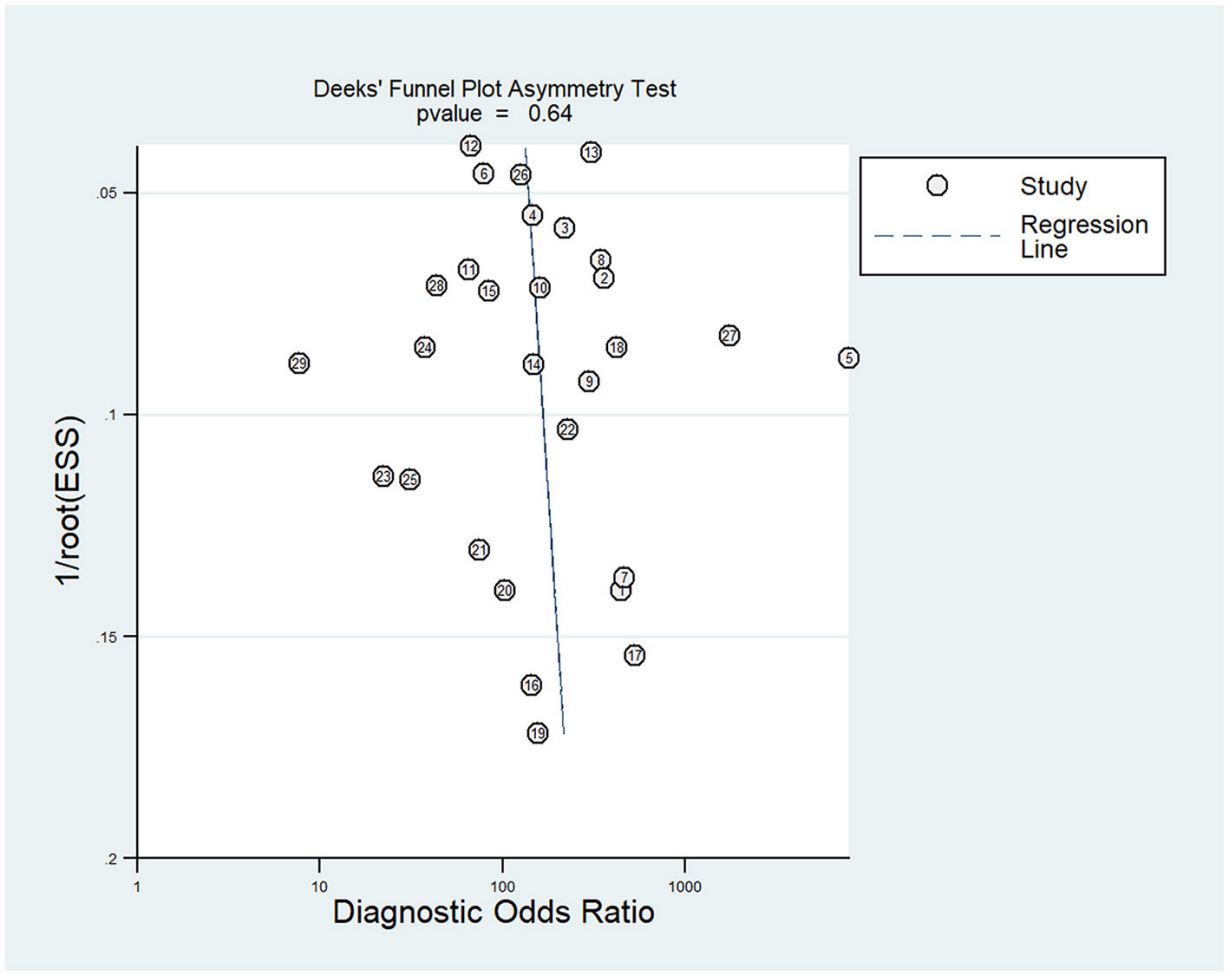
Deeks' funnel plot for assessing the publication bias.

Deek's funnel chart was used to analyze any potential publication bias. As shown in Figure 6, the funnel chart was symmetric, and *P* = 0.64 suggested that no significant publication bias existed in this meta-analysis (Figure 6).

### 3.5 Subgroup analysis

We also conducted subgroup analysis to explore the sources of heterogeneity among different populations, different numbers of included patients, and whether it was conducted in multiple centers. As shown in Table 2, our analysis showed that these variables did not have a significant impact on the pooled analysis.

**Table 2:** Subgroup analysis of diagnostic accuracies of the OSNA assay in breast cancer based on a per-node analysis.

**Subgroups**	**No. studies**	**No. patients**	**No. nodes**	**Sensitivity (95% CI)**	**Specificity (95% CI)**	**PLR (95% CI)**	**NLR (95% CI)**	**DOR (95% CI)**	**AUC (95% CI)**
**Ethnic origins**									
Caucasian	14	3,234	5,000	08.9 (0.86–0.91)	0.94 (0.93–0.94)	15.27 (9.74–23.95)	0.11 (0.08–0.18)	147.99 (72.19–303.36)	0.97 (0.96–0.99)
Asian	15	2,097	5,343	0.84 (0.81–0.86)	0.95 (0.94–0.96)	16.68 (12.47–22.31)	0.17 (0.12–0.25)	100.36 (66.97–151.20)	0.97 (0.95–0.98)
**Patient number**									
< 100	15	989	2,777	0.89 (0.86–0.91)	0.94 (0.93–0.95)	15.45 (11.34–21.04)	0.13 (0.07–0.23)	144.48 (85.31–244.69)	0.97 (0.96–0.98)
>100	14	4,342	7,566	0.85 (0.83–0.87)	0.94 (0.94–0.95)	15.79 (10.40–23.98)	0.16 (0.12–0.21)	103.38 (59.49–179.66)	0.96 (0.94–0.98)
**Center number**									
Single center	18	1,457	3,535	0.85 (0.83–0.87)	0.94 (0.94–0.95)	16.14 (10.97–23.75)	0.15 (0.11–0.22)	113.17 (66.89–191.48)	0.96 (0.94–0.98)
Multicenter	11	3,920	6,888	0.88 (0.85–0.90)	0.95 (0.94–0.96)	15.72 (11.24–22.00)	0.13 (0.09–0.20)	143.13 (76.12–269.13)	0.98 (0.96–0.99)

No., number of; 95% CI, 95% confidence interval; PLR, positive likelihood ratio; NLR, negative likelihood ratio; DOR, diagnostic odds ratio; AUC, the area under the summaryreceiver-operating characteristic curve.

### 3.6 Clinical diagnostic value

**Figure 7:**
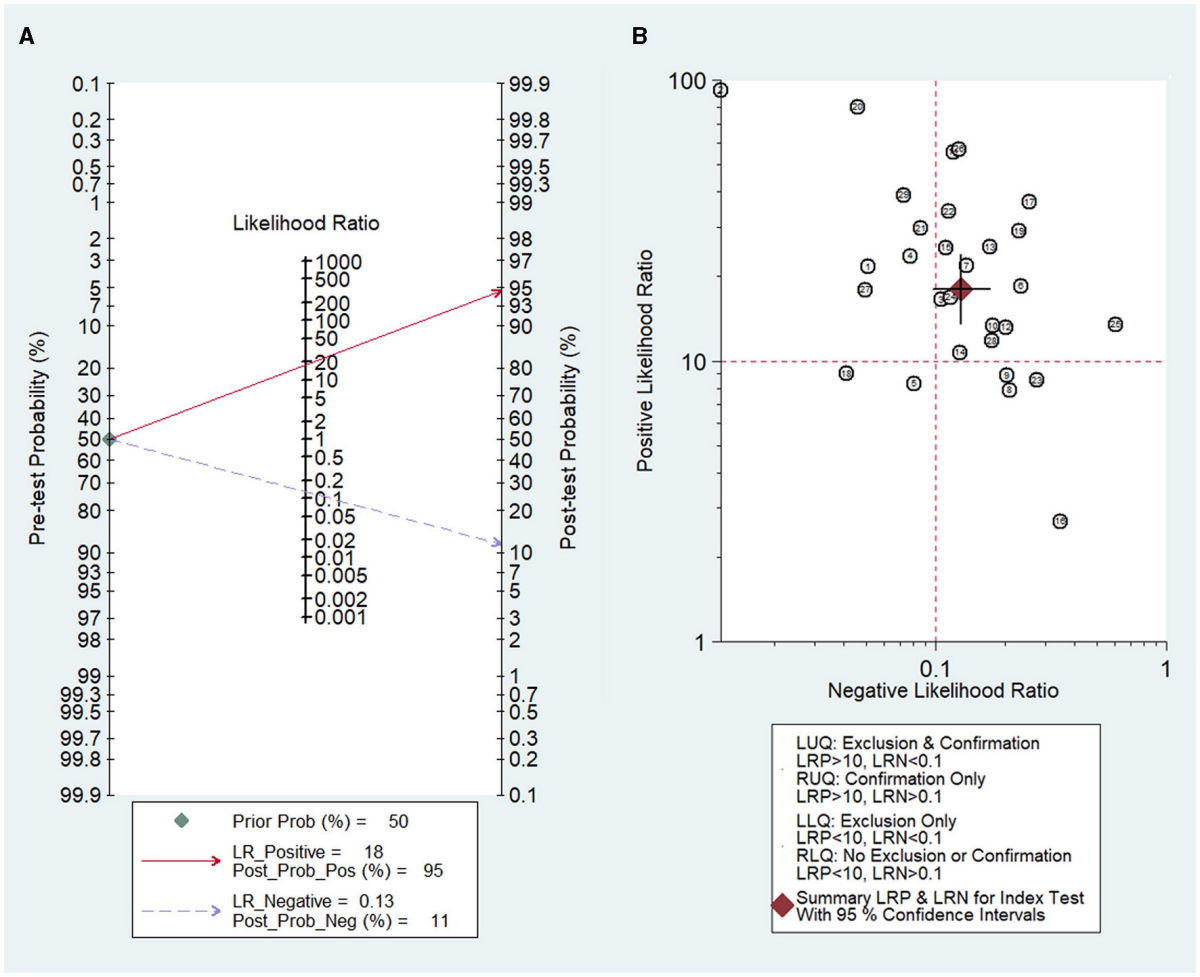
Diagnostic value of OSNA for SLN metastasis in breast cancer. **(A)** Fagan's nomogram evaluates the clinical diagnostic value of OSNA for SLN metastasis in breast cancer. **(B)** The likelihood ratio scattergram shows the different clinical significances of OSNA for SLN metastasis in breast cancer. OSNA, one-step nucleic acid amplification; SLNs, sentinel lymph nodes.

Fagan's nomogram (Figure 7A) showed a prior probability of 50%. The post-probability positive and post-probability negative were 95 and 11%, respectively. Furthermore, the likelihood ratio scattergram (Figure 7B) showed the different clinical significances of SLN metastasis in CK19-positive breast cancer. On the upper left quadrant, PLR was >10 and NLR was < 0.1, which indicated that these markers could be used to make an exclusion or confirmation diagnosis. On the upper right quadrant, PLR was >10 and NLR was >0.1, which indicated that these markers could be used to make a confirmation diagnosis only. On the lower right quadrant, PLR was < 10 and NLR was >0.1, which indicated that these markers were not able to be used to make an exclusion or confirmation diagnosis.

## 4 Discussion

The formation of distant LNM is the most lethal step in cancer progression and affects surgical decision-making, which is an important prognostic indicator in various cancer types (49). Traditional intraoperative SLN detection methods, including FS and TIC, are limited due to their low sensitivity and lack of standardized methods. An SLN biopsy combined with an intraoperative molecular-based detection of SLN metastasis using the OSNA assay provides a more standardized, objective, and reproducible whole-node assessment than the traditional pathological examination methods in breast cancer patients (16, 50). The OSNA assay can more effectively detect micrometastasis, reducing the number of false-negative histological examinations caused by the small size of micrometastases, which may not be included in any microscopical section (12). It can also avoid sampling errors and second operations due to false-negative results, thereby expediting the progression to adjuvant treatment (38). In particular, it succeeded in significantly reducing the surgical time from a mean operation length of 70.1 ± 10.5 min in the case of the FS to a mean operation length of 42.1 ± 5.1 min using OSNA (12). Therefore, it can reduce breast cancer patient healthcare costs and, correspondingly, ease the economic burden on patients. These advantages contribute to OSNA detection becoming a routine intraoperative SLN detection method during breast cancer surgery.

Although the sensitivity and specificity of the OSNA assay have been described, no pooled analysis has been conducted to evaluate the diagnostic performance of SLN metastases in breast cancer patients. In this study, we attempted to conduct a meta-analysis to quantify the diagnostic accuracy of the OSNA assay in detecting SLN metastasis in CK19-positive breast cancer patients.

A total of 29 studies, consisting of 5,331 patients with 10,343 SLNs, were included in this study. The pooled sensitivity, specificity, and AUC of the OSNA assay were 0.86, 0.94, and 0.9708, respectively, which indicated a relatively preferable diagnostic value. A previous meta-analysis based on intraoperative FS or TIC for SLNs showed that their pooled sensitivity and specificity were 0.78 and 1.00 (FS) and 0.74 and 0.98 (TIC), respectively (51). These results have also been validated by Yang et al., indicating that compared to traditional pathological examination methods, the OSNA assay seems to have better performance (52). In addition, our analysis results show that the overall diagnostic accuracy of OSNA for all cases was 0.9708. According to previous reports, the overall diagnostic accuracy of the TS and TIC detection methods was 0.9857 and 0.9837, respectively, which indicated no significant difference compared to OSNA (51). Given these potential benefits derived from the OSNA assay, the OSNA assay appears to be a useful tool in assessing SLN metastasis in CK19-positive breast cancer patients.

We also conducted subgroup analysis to explore the sources of heterogeneity among different subgroups, such as ethnic origins, patient numbers, and center numbers. There is no significant difference between different subgroups, indicating that these variables did not have a significant impact on the pooled analysis. Therefore, the OSNA assay might be quite a stable method for diagnosing SLN metastases in breast cancer. A sensitivity analysis was also performed in this study. After removing four weighted articles, the pooled specificity varied from 0.94 to 0.95, and the sensitivity, AUC value, and NLR remained unchanged. The conclusions of this study have been confirmed to be robust.

Despite our efforts to perform a systematic and comprehensive meta-analysis, there are still some limitations to our study. First, some of the included literature did not provide detailed descriptions of information, such as trial randomization, blind design, and quality control, which may affect the quality of this study. Second, the incidence and medical level were different among different countries and regions, which could affect the accuracy of the diagnosis and thus affect the results of this analysis. Third, heterogeneity among included trials is still an essential issue in this study. Moreover, this meta-analysis mainly focused on the diagnostic value of OSNA, and its prognostic value could be evaluated in future studies. Thus, the diagnostic performance and application of the OSNA assay in clinical practice still need a lot of research.

In summary, this meta-analysis provides evidence that the OSNA assay is a convenient, reliable, and standardized method for the intraoperative detection of SLN metastases in CK19-positive breast cancer patients. It provides satisfactory results in a short time, and with an easy procedure, its clinical application can benefit patients by minimizing the need for second surgeries for SLN detection.

## Data Availability

The raw data supporting the conclusions of this article will be made available by the authors, without undue reservation.
